# Presentation and course of brain metastases from breast cancer in a paranoid-schizophrenic patient: A case report

**DOI:** 10.1186/1757-1626-1-195

**Published:** 2008-09-30

**Authors:** Astrid Dalhaug, Adam Pawinski, Jan Norum, Carsten Nieder

**Affiliations:** 1 Radiation Oncology Unit, Medical Department, Nordlandssykehuset HF, 8092 Bodø, Norway; 2 Department of Oncology, University Hospital of North Norway, Tromsø, Norway; 3 Institute of Clinical Medicine, Faculty of Medicine, University of Tromsø, Tromsø, Norway

## Abstract

**Case presentation:**

This is an unusual case where a 49-year old female patient with known schizophrenia, paranoid type and a history of early-stage breast cancer, which was treated more than 6 years earlier, attempted suicide. Computed tomography and magnetic resonance imaging after this incident revealed the presence of multiple brain metastases as the first symptomatic site of recurrent cancer. Further staging lead to the diagnosis of lung, hilar and mediastinal lymph node metastases and histology confirmed estrogen receptor-positive metastatic cancer. Treatment consisted of whole-brain radiotherapy and letrozole. Twenty-one months later, the patient is in continued partial remission.

## Background

Breast cancer is one of the leading causes of brain metastases [1]. These metastases usually develop late in the course of the disease and often indicate rapid progression and short overall survival time despite various local and systemic treatment approaches [2]. Here the authors present an unusual case of a patient with schizophrenia, paranoid type (schizophrenia, PT) in whom a breast cancer relapse with multiple brain metastases was discovered following a suicide attempt.

## Case presentation

The patient (country of origin: Norway) is a caucasian female diagnosed with schizophrenia, PT at the age of 15. She had a history of alcohol and benzodiazepine abuse and symptoms of depression over several years. Several suicide attempts by intoxication were recorded before 1994. In April 1998, she had undergone mammography without pathological findings. In autumn 1999 at the age of 42 years, she had noted a small lump in her right breast, which was diagnosed as breast cancer. In November 1999, the patient was treated with modified mastectomy and axillary dissection. Histology demonstrated infiltrating ductal carcinoma grade I with associated intraductal carcinoma in situ. The invasive component measured 10 mm. The tumor was highly positive for estrogen receptor (ER) expression (95%), but less than 10% positive for progesterone receptor (PR) expression. None of the axillary lymph nodes was involved (in summary a T1 N0/7 M0 G1 ER+ PR+ breast cancer). Based on the treatment guidelines at that time, the patient did not receive any type of adjuvant treatment but follow-up examinations at regular intervals.

**Figure 1:**
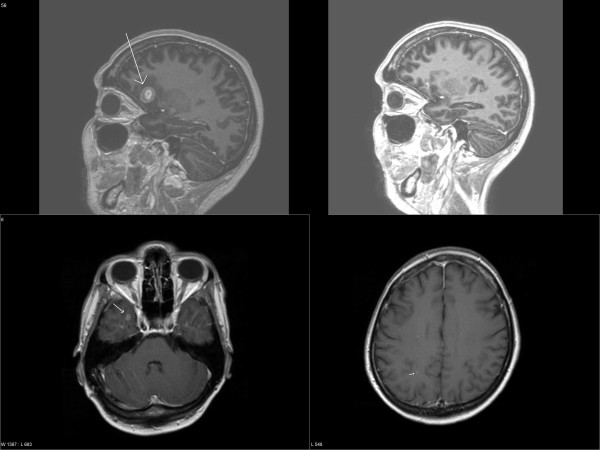
**Baseline and follow-up brain imaging**. Initial magnetic resonance imaging scans of the brain demonstrating a large frontal mass and multiple small lesions (left side and lower right, August 2006). Small residual frontal abnormality 11 months after whole-brain radiotherapy (upper right).

**Figure 2:**
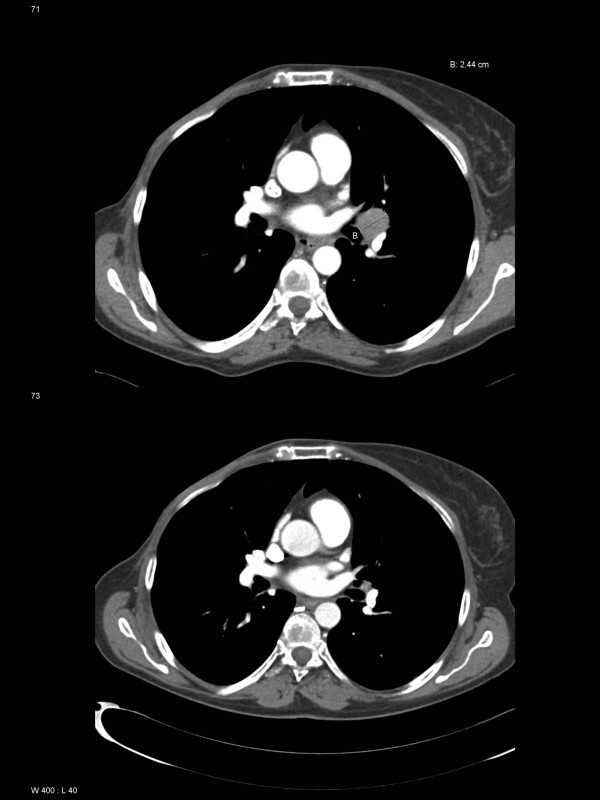
**Baseline and follow-up lung imaging**. Computed tomography scans of the chest at diagnosis of metastatic breast cancer demonstrate a left hilar mass of 2.44 cm diameter (upper image, August 2006). In November 2007, a continued partial remission was found (lower image).

In August 2006, the then 49 year-old patient attempted suicide (she tried to hang herself) while on long-term treatment with Levomepromazine and Olanzapine. After having been examined by the emergency physician, she was admitted to the hospital's orthopaedic department, where she reported irregular use of medication and increased alcohol consumption over a couple of days. She also reported about her recent nervousness and obsessive thoughts about suicide, explaining that she wanted to demonstrate her family that she was not interested in the ongoing talks about her father's assets. Although her father had passed away already one year earlier, there were still some unresolved issues around this in the family. A computed tomography scan (CT) of the brain at the day of attempted suicide showed a single supratentorial mass, while magnetic resonance imaging (MRI) revealed a total of 3 supratentorial tumors (Figure 1). Neurologic examination was unremarkable. Further staging with CT demonstrated the presence of enlarged mediastinal and left hilar lymph nodes and a small intrapulmonary lesion on the left side (Figure 2). A lymph node biopsy was taken to exclude primary lung cancer in this patient with a history of heavy smoking. Histology confirmed metastatic breast cancer, ER positive, PR negative, HER2 score 3+. Blood tests revealed pathologically elevated CA 15-3 (43 KU/liter) and lactatdehydrogenase (LDH, 234 U/liter) as the only abnormal measurements. No hypercalcemia, other distant metastases, locoregional recurrence, or contralateral breast cancer was detected. The patient was postmenopausal at that time (last menstruation in 2004).

With psychiatric support from her previous treatment team and temporary use of Zuclopenthixol, suicidality resolved and the patient tolerated the diagnostic procedures and bad news resulting from these without impairment of her condition. She was motivated to treatment and provided informed consent. Treatment consisted of whole-brain radiotherapy (WBRT, 10 fractions of 3 Gy) as inpatient on a regular oncology ward in September 2006 plus Letrozole 2.5 mg per day as continuous oral medication. No unplanned interruption of treatment was necessary. In February 2007, brain MRI showed a partial remission and the lung lesions were in minor response. CA 15-3 and LDH levels were lower, 35 KU/liter and 152 U/liter, respectively. In August 2007, these figures were 25 KU/liter and 150 U/liter, respectively. Thus, these markers were not longer pathologically elevated. MRI showed continuous partial remission (Figure 1). The last clinical follow-up examination was performed in May 2008. No potential signs of disease progression were detectable, CA 15-3 was 17 KU/liter, the lung lesion was markedly reduced in size on CT (Figure 2), and treatment with Letrozole continues. The now 51 year-old patient has a Karnofsky performance status (KPS) of 70% resulting from her known psychiatric disorder. She has no obvious late toxicity from WBRT (no detailed neurocognitive testing has been performed).

## Discussion

Patients with schizophrenia, PT have a risk of suicide that is much higher than that of the general population, e.g., 16 times higher in the study by Limosin et al. [3] and 13 times higher in the study by Osborn et al. [4]. In forensic autopsy, such incidents are rarely related to undiagnosed brain tumors, however Shiferaw et al. described a case of frontal glioblastoma diagnosed post mortem [5]. Other authors suggested that pituitary microadenoma and temporal lobe tumors might also be associated with suicide risk [6,7]. Suicide after breast cancer is also more common than suicide in the general population. In the large study by Schairer et al., the risk was elevated throughout follow-up and dependent on stage of disease [8]. The standardized mortality ratio was 1.37 and the excess absolute risk 4.1 per 100,000 person years. Cancer patients might also present with agitation, disorientation and hallucinations [9], major depression [10], and cognitive deficits [11] in the course of the disease. It was estimated that structural brain lesions were the sole cause of altered mental status in 15% of patients [9]. Whether the attempted suicide in the patient described here was caused by the presence of multiple brain metastases is hard to determine, but it appears possible. No other neurologic symptoms or signs of recurrent breast cancer were present at this time. In fact, only imaging after the attempted suicide disclosed breast cancer relapse incl. spread to the lung, hilar and mediastinal lymph nodes. Given the low-risk features at initial diagnosis, the aggressive pattern of recurrence is surprising. However, no evaluation of HER2 status was performed in 1999 when the initial diagnosis was made.

Treatment with WBRT and Letrozole was chosen after careful consideration of the patient's ability to tolerate the potential side effects of systemic treatment, her psychiatric comorbidity, the high ER expression of the metastases and the survival statistics of patients with multiple brain metastases. It was felt that more aggressive approaches would compromise the patient's compliance. WBRT plays an important role in the palliative treatment of multiple brain metastases. However, the median survival is limited to 4–6 months [2,12-14]. Performance status is the most important prognostic factor for survival [2,12,13]. Lymphopenia, which was not present in our patient, might play an additional role [12]. Regarding the 3 recursive partitioning analysis classes [15], which might be used to estimate survival, our patient belonged to class 2 because extracranial metastases were present. Median survival in class 2 breast cancer patients was reported to be 6.5 months in the study by Johansen et al. [14]. Thus, the outcome was unexpectedly favourable in the patient discussed here. Recently, it has been repeatedly reported that administration of systemic therapy after WBRT improves survival [13,16]. Also in the patient presented here, the extracranial metastases responded to endocrine therapy, which might contribute to prolongation of survival. Treatment tolerance and patient's compliance were excellent. Several salvage options incl. radiosurgery for progressive brain metastases, second-line endocrine treatment, chemotherapy, trastuzumab and lapatinib can be administered if disease progression is encountered during further follow-up [17]. With the excellent response to endocrine therapy, second line endocrine therapy should be the first choice.

## Conclusion

This case illustrates that brain metastases in patients with both cancer and schizophrenia, PT might lead to unexpected serious disease complications.

## Abbreviations

ER: estrogen receptor; PR: progesterone receptor; CT: computed tomography; MRI: magnetic resonance imaging; LDH: lactatdehydrogenase; WBRT: whole-brain radiotherapy.

## Competing interests

The authors declare that they have no competing interests.

## Authors' contributions

AD and AP treated the patient and collected the data. CN and JN drafted the manuscript. All authors read and approved the final manuscript.

## Consent

Written informed consent was obtained from the patient for publication of this case report and any accompanying images. A copy of the written consent is available for review by the Editor-in-Chief of this journal.
